# Understanding heterogeneities in mosquito-bite exposure and infection distributions for the elimination of lymphatic filariasis

**DOI:** 10.1098/rspb.2017.2253

**Published:** 2018-01-31

**Authors:** Michael A. Irvine, James W. Kazura, T. Deirdre Hollingsworth, Lisa J. Reimer

**Affiliations:** 1 School of Life Sciences, University of Warwick, Warwick, UK; 2 Institute of Applied Mathematics, University of British Columbia, Vancouver, Canada; 3 Center for Global Health and Disease, Case Western Reserve University, Cleveland, OH, USA; 4 Big Data Institute, Li Ka Shing Centre for Health Information and Discovery, University of Oxford, Oxford, UK; 5 Department of Vector Biology, Liverpool School of Tropical Medicine, Liverpool, UK

**Keywords:** lymphatic filariasis, bite heterogeneity, vector control, geospatial model, spatial heterogeneity

## Abstract

It is well known that individuals in the same community can be exposed to a highly variable number of mosquito bites. This heterogeneity in bite exposure has consequences for the control of vector-borne diseases because a few people may be contributing significantly to transmission. However, very few studies measure sources of heterogeneity in a way which is relevant to decision-making. We investigate the relationship between two classic measures of heterogeneity, spatial and individual, within the context of lymphatic filariasis, a parasitic mosquito-borne disease. Using infection and mosquito-bite data for five villages in Papua New Guinea, we measure biting characteristics to model what impact bed-nets have had on control of the disease. We combine this analysis with geospatial modelling to understand the spatial relationship between disease indicators and nightly mosquito bites. We found a weak association between biting and infection heterogeneity within villages. The introduction of bed-nets increased biting heterogeneity, but the reduction in mean biting more than compensated for this, by reducing prevalence closer to elimination thresholds. Nightly biting was explained by a spatial heterogeneity model, while parasite load was better explained by an individual heterogeneity model. Spatial and individual heterogeneity are qualitatively different with profoundly different policy implications.

## 1. Introduction

Heterogeneities in disease transmission play an important role in the epidemiology of vector-borne diseases and influence opportunities for control. The heterogeneous exposure to mosquito bites can drive vector-borne disease hotspots [1], and is a crucial factor in the optimal design of disease control intervention [2]. The degree of exposure heterogeneity can be as important as mean transmission rates in driving patterns of disease [3], but methods for measuring this heterogeneity vary and are rarely compared in the same setting [2,4,5]. It is also unclear how best to evaluate the heterogeneity of exposure within individuals in order to inform modelling and policy [6].

There are multiple levels of heterogeneity that contribute to the aggregation patterns of disease observed within a community: spatial heterogeneity, which is largely governed by ecological variation and environmental conditions [7], and individual heterogeneity, which is governed by many factors such as socioeconomic, behavioural and physiological variation among hosts [8,9]. In the context of heterogeneous exposure to mosquito bites, spatial heterogeneity may be due to landscape, rainfall, breeding site productivity, insecticide use, household size or urbanicity [1,10–13]. Individual attractiveness to mosquitoes will differ by sex, age, size and variability in human odours [14–16]. Spatial variation exists at multiple scales [9,13,17]—with different transmission dynamics between neighbouring villages and even between households [18]. These are rarely studied in the same place or through multiple measures. The transmission of lymphatic filariasis (LF), a mosquito-borne helminth infection, provides the opportunity to explore heterogeneity in bite exposure as well as in parasite burden. LF affects over 120 million people worldwide but is currently targeted for elimination. Our aim is to evaluate the multiple sources of heterogeneity which could undermine the LF elimination campaign.

Global efforts to eliminate LF through the mass distribution of anti-helminthic drugs have resulted in a large-scale reduction of prevalence [19]. However, there are numerous challenges to achieving LF elimination targets using community-wide treatments. Top-down, uniform strategies which aim for a specific intervention coverage or duration are unlikely to achieve elimination without appreciation for the significant heterogeneity driving transmission and extinction dynamics [20,21]. For LF, the target of less than 1% microfilaria (mf) prevalence set by WHO as a mark of success gives poor confidence in the probability of elimination [20]. While the recommended strategy may be sufficient in some areas [22], other areas can require many more rounds [23]. The true threshold prevalence below which transmission cannot be sustained depends on competence of the dominant vector, vector biting rates and microfilaria intensity [24]. Failure to break transmission would require community-wide mass drug administration (MDA) for the duration of the adult worm's lifespan, or direct testing and treatment, both of which may be prohibitively costly for a scaled-down LF programme. For successful elimination we require clear targets of the MDA coverage and duration needed to break transmission.

Vector control can increase the likelihood that an elimination campaign of recommended coverage and duration will achieve local elimination. The breakpoint prevalence of a vector-borne parasitic disease, below which transmission cannot be sustained, is dependent on vector biting density [25–26], so vector control will help to raise the threshold microfilaria prevalence. Supplementing MDA with vector control was recommended in countries where the burden is the heaviest [27], and evidence is mounting that vector control should be an essential component of the global elimination strategy [28]. In addition to reducing vector-borne disease transmission, vector-based interventions may also influence the spatial patterns of exposure and risk. For preventive chemotherapy vector-borne diseases such as LF, onchocerciasis and schistosomiasis, the success of community-wide coverage will be influenced by the degree of aggregation. For example, higher intervention coverage will be required in communities with highly aggregated bite risk to ensure appropriate coverage of hotspots [26]. If aggregation in biting differs significantly from village to village, a uniform strategy may underestimate the coverage required to break transmission across the implementation area (see table 1).

**Table 1.** Policy consequences for different types of heterogeneity.

Heterogeneity	High	Low
Spatial	different villages may have drastically different prevalence, one cannot be compared with the other	use of sentinel sites can be justified; reduction in one village comparable to reduction in another with the same intervention
Individual	small group of individuals highly burdened and disproportionately contributing towards ongoing infection; targeted treatment may be necessary	low variation in individuals implies blanket coverage would be effective; no small subset of population driving disease implying systematic non-adherence less of an issue

**Figure 1.**
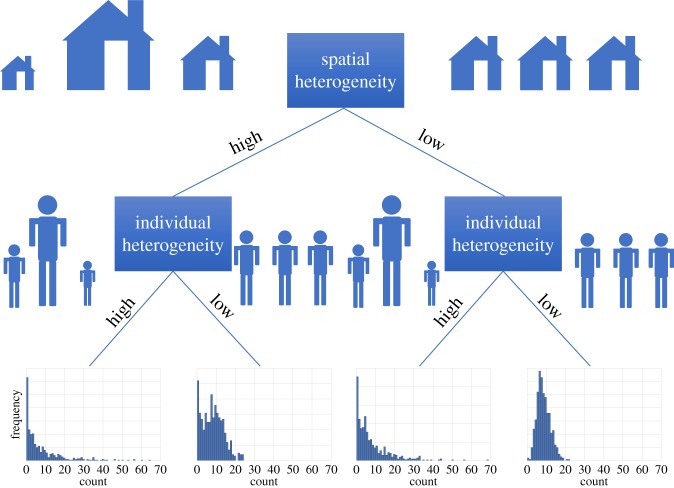
Teasing apart different types of heterogeneity. Size of houses represents relative risk in space and size of people represents relative risk in individuals. A Gaussian process is used to simulate the mean rate (e.g. biting rate) across space, with both high (left-hand side) and low (right-hand side) variance. Compounding this is the variance around the mean at each spatial location, which is referred to as intrinsic heterogeneity. Example probability distributions with a mean of 10 and high and low heterogeneity are shown across the middle. Example outcomes for the four cases are given in the bottom row. How count data is aggregated and whether there is heterogeneity among individuals (individual) and/or among space leads to qualitatively different forms of count distributions. Policy implications for each of these situations are described in table 1. (Online version in colour.)

Statistical models can be used in order to determine both spatial and individual heterogeneity within a count distribution [17] (figure 1). When individual heterogeneity is high, the count distribution is heavy-tailed and an individual's parasite count can be far from the mean. When the individual heterogeneity is low, the count distribution has a variance similar to the mean. When both spatial and individual heterogeneities are high, a highly over-dispersed distribution is produced with a greater than expected number of zeros observed compared with when the distribution is more spatially homogeneous. The result of the aggregation observed under high heterogeneity implies that an intervention that does not obtain good geographical coverage and population coverage may not be able to achieve targets in reduction or elimination. These differences in the type of heterogeneity have a profound impact on the control and elimination of parasitic disease; table 1 outlines the policy implications for each of these scenarios.

Our understanding of the sources of heterogeneity within a vector-borne disease transmission system is crucial for control and elimination because high heterogeneity is often associated with a higher basic reproduction number (*R*_0_) and a hard-to-reach threshold at which elimination can be achieved [21,29]. However, the effect of heterogeneity on disease prevalence will depend on numerous factors, including the transmission dynamics of the parasite or pathogen. Malaria parasites are cyclopropagative in the mosquito vector, while filarial worms are cyclodevelopmental with sexual reproduction occurring in the vertebrate host. Malaria transmission is highly efficient and one infective mosquito could successfully transmit malaria to multiple people. Filariasis transmission on the other hand is inefficient, requiring continuous high exposure to the infective stage larvae (up to 15 500 bites in one setting [30]) for a patent infection. Filariasis transmission models show that heterogeneous exposure results in a higher disease prevalence at low mean biting rates compared with homogeneous exposure, but this relationship changes at higher biting densities [26]. The threshold biting rate, leading to a non-zero endemic equilibrium, is significantly lower with heterogeneous biting [26]. In other words, heterogeneous exposure can sustain transmission at a comparatively lower prevalence, making it more difficult to break transmission with community-based interventions. Universal coverage of community-based interventions in a heterogeneous system may be inefficient, even leading to greater heterogeneity, and not protecting the high-risk households [31]. The spread of infection to the broader community from these households is a threat to elimination programmes and may require the integration of targeted interventions [32]. Properly implemented targeted control can result in impacts up to 4-fold higher than untargeted control [5,31].

### (a) Study aims

For elimination programmes to succeed, we must achieve the appropriate coverage, continuity and combination of interventions to break transmission and prevent resurgence. However, the approach and target coverage will depend on the aggregation of exposure and disease. It is therefore imperative to understand the impacts of heterogeneity on disease breakpoints to better tailor interventions and elimination campaigns. The aims of this study are twofold: (1) to determine what drives the heterogeneity in LF prevalence and intensity; and (2) to determine how aggregated biting patterns are influenced by vector control and the implications for LF elimination. The first aim compares the spatial relationships between breeding sites, anopheline biting rates, and infection prevalence and intensity to determine whether heterogeneity in disease status is driven by heterogeneity in either spatially dependent bite exposure or through individual variation. The second aim considers heterogeneity on a village scale by quantifying spatial aggregation of mosquito biting in five neighbouring villages before and after bed-net distribution. The fitted village biting heterogeneities are then used to parametrize an individual-based transmission model to estimate the implications of bed-net introduction on the sustainability of ongoing transmission. More broadly, this study aims to evaluate which of our standard measures and analyses of heterogeneity are most appropriate to evaluate heterogeneities which are relevant for infectious-disease control.

## 2. Methods

In order to understand the causes and effects of heterogeneity on the prevalence of LF and its underlying intensity we consider two approaches to analyse the heterogeneity of risk and infection. The first approach considers the non-spatial heterogeneity in bites and mf count by fitting these distributions by village to an over-dispersed distribution and measuring the amount of overdispersion for each fit. These fitted distributions for biting density are then applied to an individual-based model of LF transmission in order to understand how vector control impacts the ongoing transmission of LF.

The second approach considers how these indicators vary spatially and what the spatial association is between them in order to understand whether the heterogeneity in disease status or intensity is driven by spatial heterogeneity, individual heterogeneity or both. This was done by first fitting a model of individual and spatial variation to each disease outcome (mf prevalence, mf intensity and antigenic prevalence) in turn. A combined model was then used where the spatial variation is dependent on the biting density.

### (a) Study sites

Five villages in the East Sepik province of Papua New Guinea have been the focus of extensive research into filariasis epidemiology and transmission [20,33–34]. These villages received annual MDA from 1993 through 1998, with no further interventions until long-lasting insecticidal nets (LLINs) were distributed in August 2009. Self-reported LLIN use ranged 75–90% [35].

### (b) Infection prevalence

Antigen prevalence and microfilaria prevalence were measured in these communities in 2008 as part of the post-MDA evaluation [35]. This was done by BinaxNow filariasis antigen test and by microscopic evaluation of 1 ml filtered venous blood, collected at night (21.00–03.00). The age and sex of participants were recorded as well as the time of blood collection. The GPS coordinates of all households were recorded.

### (c) Mosquito collection

Mosquitoes were collected monthly by the human landing catch method from July 2007 through July 2010 as described by Reimer *et al.* [35]. Villages were divided into four quadrants and houses were chosen from each quadrant every month for even sampling across the village. Mosquitoes were collected in the front of the house from July 2007 through July 2010. Quarterly collection continued in Nanaha and Yauatong through December 2011. The total collection effort ranged between 40 and 48 collection nights. All host-seeking anophelines were included in the density summary. *Anopheles punctulatus* comprised the majority of mosquitoes collected; additional members of the *An. punctulatus* group included *An. koliensis*, *An. hinesorum*, *An. farauti* 4 and *An. farauti s. s.* [36].

All temporary and permanent breeding sites were geolocated. These breeding sites were further categorized as confirmed or potential depending on the presence of anopheline larvae at the time of the survey.

### (d) Non-spatial modelling

To determine the heterogeneity at each village before and after the distribution of bed-nets, a negative-binomial distribution was fitted to both the mf count and bite data, parametrized by the mean *m*, and the heterogeneity parameter *k*. Here, a smaller *k* indicates a more over-dispersed distribution and a higher *k* indicates a less dispersed or more Poisson-like distribution. For a count *n*, the probability distribution is defined as2.1
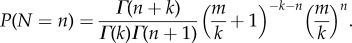
The negative-binomial distribution was fitted to village-level count data using a maximum-likelihood approach. For the nightly mosquito catches, the data were stratified before and after LLINs were distributed as a further measure of the impact of vector control on heterogeneity.

### (e) Infection transmission model

In order to understand the fitted heterogeneity *k* and mean biting density before and after the introduction of bed-nets in the context of disease transmission, the results were compared with an established model of LF transmission, TRANSFIL [26]. The model is a multi-scale stochastic simulation of individuals with worm burden, microfilaraemia and other demographic parameters relating to age and risk of exposure. Humans are modelled individually, with their own male and female worm burden. The density of mf in the peripheral blood is also modelled for each individual and is dependent on the number of female worms. The total mf density in the population contributes towards the instantaneous density of L3 larvae in the human-biting mosquito population. This density combined with the mosquito biting rate and an intrinsic factor that varies between individuals determines the probability of an individual being infected with a new adult worm. See [26] for a full model description.

### (f) Spatial modelling

In order to determine how much spatial variation and individual variation contribute towards differences in disease status between individuals a number of geospatial models were implemented. These models take into account distance to breeding sites, anopheline biting rates, and infection prevalence and intensity. The first group of models compare the measured disease statuses dependent on a random spatially varying risk. The second group of models combine together the biting density and disease status, by assuming that spatial variation in status is determined by the biting density alone.

*Gaussian process.* The underlying spatial variability (in mosquito bites, mf intensity, parasitaemia and antigenaemia) is modelled using a Gaussian process *S*(*x*_*i*_) for each spatial location *x*_*i*_. A Gaussian process describes the spatial relationship between different spatial locations and is defined as, given a set of locations {*x*_*i*_}, the probability of observing the set {*S*(*x*_*i*_)} is a multivariate Gaussian probability with zero mean and a defined covariance function. For flexibility and computational reasons, a Matérn covariance function was used, which is defined as2.2
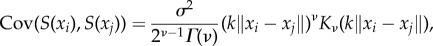
where *K*_*ν*_ is a modified Bessel function of the second kind and order *ν* > 0. The Matérn covariance function has three free parameters which control the marginal variance, the distance of spatial correlation and the sharpness of the function. These parameters can be combined to give the distance at which there is less than 10% correlation between two points, which is given as 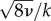. This is referred to as the practical range.

*Modelling distance to breeding sites.* A second set of data includes the spatial locations of sites that may contribute to mosquito breeding. These sites include pig houses, creeks, gardens and garden houses. These data and the household bite data do not match up in terms of their geolocations. In order to circumvent this problem, we may instead use the minimum distance to a breeding site as a covariate to inform the models. All breeding sites are assumed to be equivalent whether they contained anopheline larvae or not, as there are only a limited number of sites, so as to increase power of the covariate.

*Random walk latent model for breeding site distance.* The relationship between minimum distance to breeding sites and the number of bites was found to be nonlinear (see the electronic supplementary material). In order to capture the full complexity observed in this relationship a more general functional form of the distance *d*_*j*_ was used, i.e. *f*(*d*_*j*_). For this functional form, distances were split into a discrete lattice of points. Each lattice point *k* then has a corresponding coefficient *x*_*k*_ related to the nightly bites through the log intensity in the negative binomial. The assumed model was a random walk of length one, i.e. *x*_*k*+1_∼*N*(*x*_*k*_, *σ*^2^). The fitted function *f*(*d*_*j*_) then returns the coefficient *x*_*k*_ corresponding to the nearest lattice point to the actual distance *d*_*j*_.

*Infection status and mosquito catch models*. Both the infection status of each individual and the mosquito nightly bites were modelled separately using a generalized linear model including fixed effects for each observed variable (electronic supplementary material, figure S1). The general form of the model is, given a set of observations {*y*_*i*_} at spatial locations {*x*_*i*_}, the outcomes *y*_*i*_ have the distribution2.3
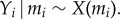
For a general random variable *X*, with underlying mean *m*_*i*_. The mean is also a random variable with the structure2.4
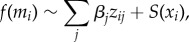
where *f* is a link function transforming the mean from the positive numbers to the entire real line, *z*_*ij*_ is the *j*th covariate for the *i*th data-point and *β*_*j*_ is the regression coefficient for the *j*th covariate, which also includes an intercept. *S*(*x*_*i*_) is the Gaussian process as previously defined.

For both the mosquito bite model and the mf count model, the random variable *X* that the observations are drawn from is assumed to be negative binomial with aggregation (heterogeneity) parameter *k*. The microfilaraemia and antigenaemia models have observations that are either positive (1) or negative (0), and hence the observations are assumed to be drawn from a Bernoulli random trial, i.e.2.5
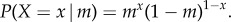
The link function for the negative-binomial models was taken to be log and the link function for the Bernoulli models was taken to be logit.

The fixed effects for each model considered for the infection status models were the distance to breeding site, age of the individual and the sex of the individual. For the mosquito bite model, the covariates included were the presence or absence of bed-nets and the distance to breeding site.

*Combined model.* The models for the infection status were generalized to have this Gaussian process derived from the mosquito catch data, rather than including an independent spatial Gaussian process. The two-level hierarchical structure incorporates both the disease status for an individual *Y*_*i*_ and the bites at household locations *B*_*i*_. Both observations are drawn from their own random variables *X* and *Y* , with underlying means *m*_*i*_ and *n*_*i*_, respectively. The random variables are connected through a linear model structure of these two means with their covariates combined with a GP fitted to the transformed bite means. This GP is related to the infection status mean through the coefficient *η*, which measures the dependency of the infection status mean on the underlying spatial distribution of bites conditioned on the fixed effects of the bites. Mathematically, the model is defined as2.6
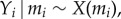
2.7
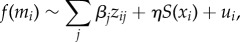
2.8
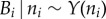
2.9
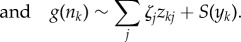
Here the underlying Gaussian process is assumed to capture the distribution of bites, and is fitted to both the bites and infection status simultaneously. *η* gives the strength of the dependency on the underlying spatial structure of bites on infection status and *u*_*i*_ is a random effect with variance *σ*^2^_*u*_ used to capture the variation observed in the infection status that is not captured by the bite data.

*Model fitting.* The model fitting was performed using the R-INLA package [37]. This implements an integrated nested Laplace approximation (INLA) method, which is a faster alternative to Markov chain Monte Carlo (MCMC) for certain classes of models. The package also approximates the Gaussian process as a Gaussian Markov random field (GMRF), which approximates the continuous space used in GP, by a discretization of space using a triangulation based on the spatial location of the data points [38–39]. The spatial model fitting also provides an estimate of the underlying mean and variation across space.

*Model comparison.* In order to systematically compare the spatial and non-spatial variants of the mosquito nightly catches (bites) and mf, the Akaike information criterion (AIC) was used to assess which model produces a better fit to the data [40]. This is defined as2.10
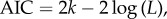
where *L* is the maximum likelihood of the model and *k* is the number of model parameters. Two AIC were compared by taking the difference of the two.

## 3. Results

**Figure 2.**
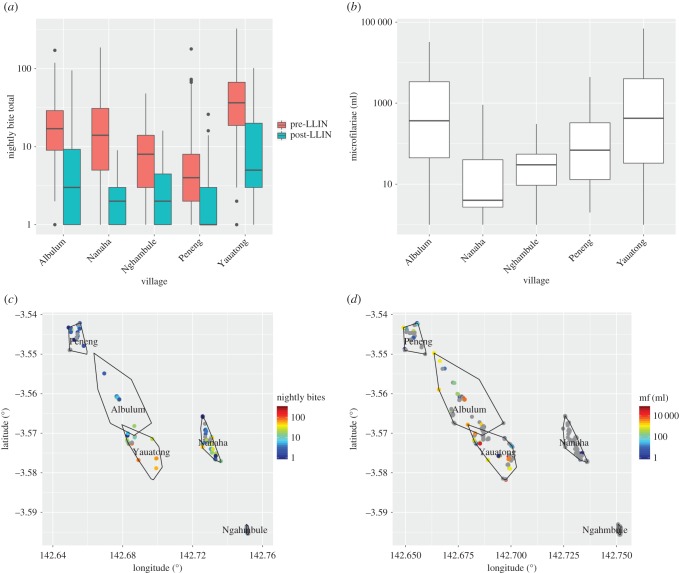
Heterogeneity data used in the study. (*a*) Nightly bite total by village. (*b*) Distribution of mf count by village. (*c*) Spatial distribution of bites with colours on a log scale (distance approx. 13 × 6 km). (*d*) Spatial distribution of mf intensity with colours on a log scale (distance approx. 13 × 6 km). The spatial data indicates Yauatong is a hotspot for biting, and Albulum and Yauatong are hotspots for the presence and intensity of mf. Grey values in (*c*,*d*) indicate zero values for the nightly bites and mf concentration, respectively. (Online version in colour.)

The LF antigen prevalence, microfilaria prevalence and intensity were collected from all consenting community members (*n* = 1046 individuals). Nightly biting data were collected from 170 households with 2180 sampling nights total (figure 2).

### (a) Heterogeneity within villages

**Figure 3.**
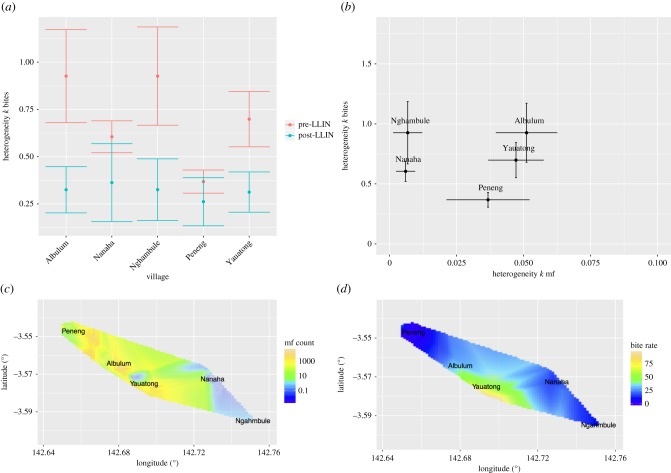
Heterogeneity of disease and mosquito bites at village and spatial levels. (*a*) Comparison of the heterogeneity as measured from the negative-binomial distribution before and after bed-nets. (*b*) Bites pre-LLIN compared with heterogeneity in mf count among individuals. The maximum-likelihood estimates for each are given as points with 95% CI given as error bars. (*c*,*d*) Spatial fits of hierarchical model for (*c*) mf count and (*d*) nightly bite rate.

A negative-binomial distribution was fitted independently to bite counts in each village before and after the introduction of bed-nets (LLIN) (figure 3*a*). There is significant variation in the heterogeneity between villages pre-LLIN. A reduction in *k*, corresponding to an increase in heterogeneity, is observed across most villages. Where this reduction is significant is in Albulum, Nananha, Ngahmbule and Yauatong. For Peneng, there is observed a reduction in the maximum-likelihood estimate of *k*, although the confidence intervals of the estimate overlap.

To determine whether aggregated biting patterns were associated with an aggregated parasite population, we compared the pre-LLIN bite rate heterogeneity with the mf count heterogeneity (figure 3*b*). There was observed a large amount of variation in the mf count heterogeneity, with Nanaha and Nghambule less than 0.0125, and Peneng, Yauatong and Albulum with heterogeneity greater than 0.03. The heterogeneity in the mf counts is significantly greater than the bites in all cases. There is a positive relationship between the two heterogeneities, although the correlation is extremely weak (correlation coefficient 0.012).

### (b) Impact on elimination

**Figure 4.**
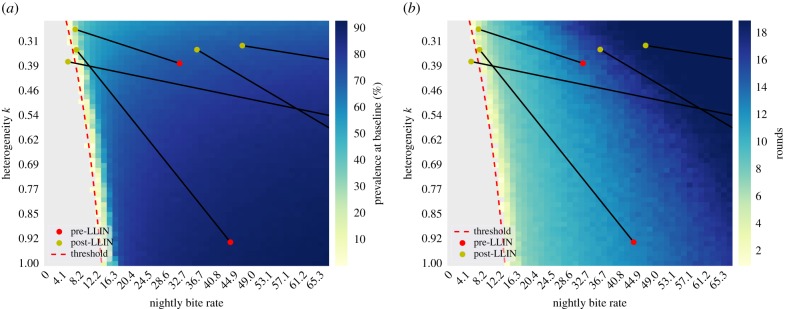
Comparison between the theoretically predicted (*a*) prevalence at baseline and (*b*) number of rounds until reaching pre-TAS levels for varying heterogeneity and vector-to-host ratio. The red and yellow dots represent the fitted bite data before and after bed-nets, respectively. The theoretical threshold for the break in transmission is shown as a red dotted line. LLINs caused a reduction in biting density and an increase in heterogeneity, which is associated with fewer rounds of MDA to cross the threshold.

The change in the vector-to-host ratio and the heterogeneity in bites after the intervention of bed-nets was explored using the stochastic model of LF transmission TRANSFIL [26]. The number of rounds to 1% microfilaraemia (which is used as an assessment for halting MDA [41]) and the prevalence at baseline before the start of any intervention were calculated across a range of bite heterogeneity and vector-to-host ratio values (figure 4). The threshold at which transmission is broken and infections are no longer sustained in the population was also calculated from these simulations. For increased heterogeneity, a smaller vector-to-host ratio, and therefore mean monthly bite rate, can sustain transmission. However, for decreased heterogeneity the vector-to-host ratio required to sustain infection increases. The effect of bed-nets can be seen to both reduce the vector-to-host ratio as well as increase the heterogeneity of bites, although for the villages in the study, the reduction in the vector-to-host ratio more than offsets the increased heterogeneity. Figure 4*b* highlights the number of rounds required to pre-TAS without any prior intervention. For high heterogeneity many more rounds would be required than for the equivalent bite rate at smaller heterogeneity. The impact of bed-nets can clearly be seen to rapidly reduce the number of rounds required in each village. The range in predicted rounds between villages is also large; this is, however, reduced by the introduction of bed-nets.

### (c) Spatial modelling

With a weak but positive association between heterogeneous biting and heterogeneous infection, we sought to determine whether this pattern could be interpreted due to spatial variation. Spatial heterogeneity was therefore explored for both the bite distribution and distribution of mf count, microfilaraemia and antigenaemia. The fixed effects considered for each of the infection status spatial models were sex of the individual, age of the individual and the minimum distance to breeding site. For the spatial mosquito catch model, both the presence of LLIN and distance to breeding site were considered as fixed effects. There was found to be no significant seasonal trend in bites, and hence month at which bite survey was conducted was not included. There was also found to be no significant trend in the time at which bleeds were taken, and hence this was also not included.

For the infection status models, age was found to be statistically significant in all cases (*p* < 0.001), although with a small effect in all cases. Sex of the individual was found to only be marginally significant (*p* = 0.15, 0.05, 0.02 for microfilaraemia, antigenaemia and mf count, respectively), with males having an increased risk of microfilaraemia, antigenaemia and mf count. The distance to breeding site was not found to be significant for any of the cases.

For the spatial mosquito catch model the presence of LLIN was found to be statistically significant, with the presence of bed-nets decreasing the coefficient by 76%. The distance to breeding site was both not statistically significant and had a very small effect compared with the intercept. The overall calculated *k* for the infection status mf count model was estimated at 0.05, with a standard deviation of 0.0043 and the *k* for the bites model was estimated to be 0.73 with a standard deviation of 0.035. The overall heterogeneity in both cases was, therefore, broadly in keeping with the estimates of the villages separately, where the mf count heterogeneity varied between 0.05 and 0.01 and the heterogeneity of bites for the villages where the mf surveys were conducted was between 0.9 and 0.3.

The fitted spatial model also provides an estimate of the mean intensity for infection status and bites across space (figure 3*c*,*d*). The estimated bite rate intensity is distributed around Yauatong, with a maximum bite rate of around 60 (figure 3*d*). This decreases smoothly to zero out towards Ngahmbule to the southeast and Peneng to the northwest. By contrast, both the prevalence of antigen and mf have the highest intensity around Peneng in the northwest, with a smooth decrease down towards Ngahmbule. Both prevalence spatial patterns exhibit different underlying intensity. There is a significant increase in antigenaemia around Yauatong, with a similar, but less pronounced increased risk of microfilaraemia. The uncertainty in prevalence of mf is high for Peneng, Albulum and Yauatong in the northwest, and small in the southwest (near Yauatong), where the mean prevalence is around 40%. The mf count mean is more varied than for the other distributions, with high-intensity areas around the southern part of Yauatong, Albulum and the southeast perimeter of Peneng. Ngahmbule and Nanaha have the lowest mf count matching with the lowest villages for antigenaemia and bite rate. Peneng has high levels of antigenaemia and mf count; however, it is the lowest for bites.

In order to understand the difference in spatial scale between infection status and bites, the fitted covariance structure from the mf count spatial model was compared with the structure from the bite model (electronic supplementary material, figure S1). The practical range for the mf spatial correlation was smaller than the range for the bites (0.014° to 0.008° or ≈1.5 km to ≈0.9 km). The difference in the marginal (non-spatial) variance is also great, with the variance for the mf field *σ*^2^ = 38 and the variance for the bites *σ*^2^ = 5. There was found to be no significant difference in the fitted *κ* (sharpness of covariance) between the mf and bite count.

The change in AIC between the spatial and non-spatial model for mf was 1308.26, whereas for the bites model was −352.03 indicating the bite distribution is better explained by a spatial model and mf count is better explained by a non-spatial model.

*Combined model.* In the final analysis, the bite data and infection status data were combined to produce a bite-count-dependent spatial field that is also used to predict the distribution of mf count, antigenaemia and microfilaraemia separately. The fixed effects used in the first spatial analysis were kept for the combined model. All fixed effects were found to have a similar strength and significance as in the separate models. The coefficient that described the strength of the bite rate spatial field on the outcome of the disease status spatial distribution was also calculated. These coefficients were found to be significant for mf count, microfilaraemia and antigenaemia. The largest dependency was for mf count (1.71 (1.38, 2.06)), with antigenaemia the second strongest (1.17 (1.13, 1.21)) and microfilaraemia the weakest (1.00 (0.96, 1.00)). A spatially independent random-effects term was also included in the model to account for all variation not already accounted for by the fixed effects or the spatial distribution produced by the bites model. These were found to be negligible in all cases.

## 4. Discussion

The primary aim of the study was to determine whether heterogeneous biting activity drives the observed heterogeneity in LF prevalence and intensity. We observed a much more complex picture than previously expected, with heterogeneity being driven by both spatial biting patterns and individual processes. The secondary aim was to determine how aggregated biting patterns are influenced by vector control and determine the implications for LF elimination. Combining a statistical and modelling approach, we demonstrate that vector control increases heterogeneity while also uniformly reducing biting. This resulted in decreased variability in the predicted number of years required to achieve elimination between neighbouring villages in Papua New Guinea.

Heterogeneity poses numerous challenges to global elimination programmes that rely on broad-scale mapping to inform distribution of community-wide interventions. Heterogeneities in exposure and infection are well-known drivers of persistent disease transmission. Diseases such as LF have complex ecological interactions that can lead to threshold behaviour, where sustained transmission is dependent on biting density or parasite load [24]. The basic reproduction number *R*_0_ is expected to be greater under heterogeneous biting [42]. The probability of re-introduction of a disease in a fully susceptible population can also have a nonlinear relationship with the basic reproductive number of the infection [43]. The potential for transmission can be dependent on heterogeneous exposure (some people bitten by mosquitoes more than others), poor mixing (non-random contacts between hosts and mosquitoes) and finite population sizes (each host can contribute at most one new infection towards the population total) [44].

Heterogeneities in infection can complicate disease surveillance programmes since public health infection mapping is usually performed at village/town level, while interventions are often implemented at a broader administrative level. Data aggregated by population can hide the true patterns, which are more apparent when data are considered in a spatially explicit fashion. It is therefore imperative to understand the relationship between the underlying heterogeneity for these scales and how this heterogeneity impacts the efficacy of interventions [17]. Spatial and individual heterogeneity should be considered in order to ensure implementation policy is appropriate to local transmission and epidemiology (table 1).

Individual heterogeneity of infection in a population reduces the likelihood that community-wide interventions are protecting the highly exposed. As a result there is a lower threshold mf prevalence required to break transmission and a greater likelihood that high-density infections in a few individuals can seed new infections (figure 1). Spatial heterogeneity can also conflate an elimination campaign, as there may be regions of high disease burden adjacent to regions with low rates of transmission. This poses a significant challenge when sentinel and spot-check sites are used to determine the prevalence for an entire region for the purposes of implementation [45]. This may lead to limited resources being wasted on MDA distribution in villages that have lower than threshold prevalence, while possibly missing areas that will require a longer duration of MDA to break transmission.

We observed a strong spatial correlation between biting density and antigenaemia, which captures current or prior presence of adult worms, including amicrofilaraemic infections. While biting density was also significantly associated with microfilaria intensity, this association was weaker than for either antigen or microfilaria prevalence, indicating a weak relationship between high exposure to mosquito bites and intensity of infection. Although the distance to breeding site was not statistically significant for infection status or bites, there is a stronger mean effect from the bite counts than from the infection status. The distance to breeding site would naturally be more associated with biting density, whereas infection status is more strongly dependent on other factors, hence the weaker regression effect. As current microfilaria intensity is the result of fecund adult worms that have established after years of exposure to infective bites, long-term changes in the mosquito population may not be taken into account from the current distribution of breeding sites. Previous studies in these study villages have shown that high density infections are associated with reduced strain variation, and are not necessarily due to multiple adult worms [46]. Host immunity or *W. bancrofti* strain fecundity probably play a greater role in the intensity of microfilariae.

In our study heterogeneity in bite exposure varied substantially from village to village before the LLIN distribution, and this was associated with wide-ranging predictions on the number of rounds of MDA required to break transmission. Heterogeneity increased significantly after the introduction of LLINs in all villages except the one village with very low pre-LLIN biting rates, resulting in a very similar heterogeneity parameter across the five villages. The greater heterogeneity observed post-LLIN in all communities is associated with a transmission threshold at a lower mean biting rate. In this particular transmission system, the reduction in vector density caused by the LLIN distribution compensated for this change in threshold biting rate. However, it does highlight the extreme importance of considering heterogeneity in elimination strategies, because changes in heterogeneity cause elimination targets to move. Globally there are 54 countries engaging in preventive chemotherapy for the elimination of LF [47], and each of these countries will need to decide when to stop MDA and switch to long-term surveillance. That decision will be made based on the available evidence that microfilaria prevalence has fallen below 1% in sentinel villages, but there is a risk that the minimum duration of MDA will differ significantly between neighbouring villages.

Statistical models can help shed light on the complex factors that contribute towards heterogeneity in disease-burden and transmission [48]. While heterogeneity in the vector population can lead to a particular aggregated exposure, there is an assumption that aggregated exposure leads to an aggregated burden of infection. In transmission models for macroparasites, there is usually an explicit distribution of risk across individuals in the same community, but it is often parametrized against the resulting distribution of infection burden rather than vector data [26,49,50]. Here we have demonstrated that factors including individual and spatial heterogeneity can all contribute towards the perceived variation in the aggregated distribution (figure 1). Although here bites are measured at the household as opposed to the individual-level, these results suggest that modelling should more explicitly take into account other aspects that lead to the final distribution, such as strength of infection-blocking immunity. However, we acknowledge that variation in infection burden has been much more frequently measured than variation in vector biting rates, due to understandable practical challenges, and therefore this may be the best way to proceed in the absence of more vector data. In other vector-borne disease, heterogeneity in exposure is rarely explicitly included in transmission models, despite a number of measurements and theoretical studies highlighting its importance [1,3,5,7]. Our study once again demonstrates the likely impact of these heterogeneities, and the need for more epidemiological and entomological studies performed at the same time and in the same place. While these data are challenging to interpret, larger studies would allow us to identify the right correlates of current transmission rates and the likely impact of control.

## 5. Conclusion

Understanding sources of heterogeneity is important both in disease modelling and ultimately in the control and elimination of a disease. We have comprehensively demonstrated here that individual and spatial heterogeneity can impact disease prevalence and intensity in different ways, and has direct implications to policy. There are many logistical and financial challenges to sustaining long-term MDA campaigns in a setting like Papua New Guinea, where communities are hard to reach and departments of health have competing priorities. The risks of resurgence if programmes fail to break transmission thresholds would compromise the gains already made by global elimination efforts. Therefore, knowledge of the degree of heterogeneity is necessary to understand where transmission thresholds lie, and understanding the sources of heterogeneity is essential to designing and delivering interventions with the greatest chance of success.

## Supplementary Material

Supplementary information

## Data accessibility

This article has no additional data.

## References

[1] Bousema T, Griffin JT, Sauerwein RW, Smith DL, Churcher TS, Takken W, Ghani A, Drakeley C, Gosling R 2012 Hitting hotspots: spatial targeting of malaria for control and elimination. PLoS Med. 9, e1001165 (10.1371/journal.pmed.1001165) 22303287 PMC3269430

[2] Woolhouse MEJ *et al.* 1997 Heterogeneities in the transmission of infectious agents: implications for the design of control programs. Proc. Natl Acad. Sci. 94, 338–342. (10.1073/pnas.94.1.338) 8990210 PMC19338

[3] Ross A, Smith T 2010 Interpreting malaria age-prevalence and incidence curves: a simulation study of the effects of different types of heterogeneity. Malar. J. 9, 132 (10.1186/1475-2875-9-132) 20478060 PMC2888834

[4] Lloyd-Smith JO, Schreiber SJ, Kopp PE, Getz WM 2005 Superspreading and the effect of individual variation on disease emergence. Nature 438, 355–359. (10.1038/nature04153) 16292310 PMC7094981

[5] Smith DL, McKenzie FE, Snow RW, Hay SI 2007 Revisiting the basic reproductive number for malaria and its implications for malaria control. PLoS Biol. 5, e42 (10.1371/journal.pbio.0050042) 17311470 PMC1802755

[6] Brooker S, Alexander N, Geiger S, Moyeed RA, Stander J, Fleming F, Hotez PJ, Correa-Oliveira R, Bethony J 2006 Contrasting patterns in the small-scale heterogeneity of human helminth infections in urban and rural environments in Brazil. Int. J. Parasitol. 36, 1143–1151. (10.1016/j.ijpara.2006.05.009) 16814294 PMC1783908

[7] Midega JT *et al.* 2012 Wind direction and proximity to larval sites determines malaria risk in Kilifi District in Kenya. Nat. Commun. 3, 674 (10.1038/ncomms1672) 22334077 PMC3292715

[8] Maizels RM, Bundy DAP, Selkirk ME, Smith DF, Anderson RM 1993 Immunological modulation and evasion by helminth parasites in human populations. Nature 365, 797–805. (10.1038/365797a0) 8413664

[9] Smith DL, Dushoff J, Snow RW, Hay SI 2005 The entomological inoculation rate and *Plasmodium falciparum* infection in African children. Nature 438, 492–495. (10.1038/nature04024) 16306991 PMC3128496

[10] Walker M, Winskill P, Basáñez M-G, Mwangangi JM, Mbogo C, Beier JC, Midega JT 2013 Temporal and micro-spatial heterogeneity in the distribution of *Anopheles* vectors of malaria along the Kenyan coast. Parasit. Vectors 6, 311 (10.1186/1756-3305-6-311) 24330615 PMC3843567

[11] Frank C *et al.* 2016 Spatial heterogeneity of malaria in Ghana: a cross-sectional study on the association between urbanicity and the acquisition of immunity. Malar. J. 15, 84 (10.1186/s12936-016-1138-4) 26867774 PMC4751679

[12] Acevedo MA, Prosper O, Lopiano K, Ruktanonchai N, Caughlin TT, Martcheva M, Osenberg CW, Smith DL 2015 Spatial heterogeneity, host movement and mosquito-borne disease transmission. PLoS ONE 10, e0127552 (10.1371/journal.pone.0127552) 26030769 PMC4452543

[13] Favier C, Schmit D, Müller-Graf CDM, Cazelles B, Degallier N, Mondet B, Dubois MA 2005 Influence of spatial heterogeneity on an emerging infectious disease: the case of dengue epidemics. Proc. R. Soc. London B: Biol. Sci. 272, 1171–1177. (10.1098/rspb.2004.3020) PMC1559809 16024379

[14] Muirhead-Thomson RC 1951 Distribution of anopheline mosquito bites among different age groups. Br. Med. J. 1, 1114 (10.1136/bmj.1.4715.1114) 14830852 PMC2068913

[15] Burkot TR 1988 Non-random host selection by anopheline mosquitoes. Parasitol. Today 4, 156–162. (10.1016/0169-4758(88)90151-2) 15463075

[16] Smallegange RC, Takken W 2010 Host-seeking behaviour of mosquitoes: responses to olfactory stimuli in the laboratory. In *Olfaction in vector-host interactions*, vol. 2 (eds W Takken, BGJ Knols), pp. 143–180. Wageningen, The Netherlands: Wageningen Academic Publishers.

[17] Moraga P *et al.* 2015 Modelling the distribution and transmission intensity of lymphatic filariasis in sub-saharan africa prior to scaling up interventions: integrated use of geostatistical and mathematical modelling. Parasit. Vectors 8, 1–16. (10.1186/s13071-015-1166-x) 26496983 PMC4620019

[18] Bejon P *et al.* 2010 Stable and unstable malaria hotspots in longitudinal cohort studies in Kenya. PLoS Med. 7, e1000304 (10.1371/journal.pmed.1000304) 20625549 PMC2897769

[19] Ramaiah KD, Ottesen EA 2014 Progress and impact of 13 years of the global programme to eliminate lymphatic filariasis on reducing the burden of filarial disease. PLoS Negl Trop. Dis. 8, e3319 (10.1371/journal.pntd.0003319) 25412180 PMC4239120

[20] Michael E, Singh BK 2016 Heterogeneous dynamics, robustness/fragility trade-offs, and the eradication of the macroparasitic disease, lymphatic filariasis. BMC Med. 14, 1 (10.1186/s12916-016-0557-y) 26822124 PMC4731922

[21] Gambhir M, Bockarie M, Tisch D, Kazura J, Remais J, Spear R, Michael E 2010 Geographic and ecologic heterogeneity in elimination thresholds for the major vector-borne helminthic disease, lymphatic filariasis. BMC Biol. 8, 22 (10.1186/1741-7007-8-22) 20236528 PMC2848205

[22] Ramzy RMR, El Setouhy M, Helmy H, Ahmed ES, Elaziz KMA, Farid HA, Shannon WD, Weil GJ 2006 Effect of yearly mass drug administration with diethylcarbamazine and albendazole on bancroftian filariasis in Egypt: a comprehensive assessment. Lancet 367, 992–999. (10.1016/S0140-6736(06)68426-2) 16564361

[23] Rebollo MP, Mohammed KA, Thomas B, Ame S, Ali SM, Escalada AG, Cano J, Bockarie MJ 2015 Cessation of mass drug administration for lymphatic filariasis in Zanzibar in 2006: was transmission interrupted? PLoS Negl. Trop. Dis. 9, e0003669 (10.1371/journal.pntd.0003669) 25816287 PMC4376862

[24] Gambhir M, Michael E 2008 Complex ecological dynamics and eradicability of the vector borne macroparasitic disease, lymphatic filariasis. PLoS ONE 3, e2874 (10.1371/journal.pone.0002874) 18716676 PMC2518518

[25] Duerr HP, Raddatz G, Eichner M 2011 Control of onchocerciasis in Africa: threshold shifts, breakpoints and rules for elimination. Int. J. Parasitol. 41, 581–589. (10.1016/j.ijpara.2010.12.009) 21255577

[26] Irvine MA, Reimer LJ, Njenga SM, Gunawardena S, Kelly-Hope L, Bockarie M, Hollingsworth TD 2015 Modelling strategies to break transmission of lymphatic filariasis-aggregation, adherence and vector competence greatly alter elimination. Parasit. Vectors 8, 1–19. (10.1186/s13071-015-1152-3) 26489753 PMC4618540

[27] World Health Organization. 2012 *Accelerating work to overcome the global impact of neglected tropical diseases: a roadmap for implementation*. Geneva, Switzerland: WHO.

[28] Bockarie MJ, Pedersen EM, White GB, Michael E 2009 Role of vector control in the global program to eliminate lymphatic filariasis. Ann. Rev. Entomol. 54, 469–487. (10.1146/annurev.ento.54.110807.090626) 18798707

[29] Koella JC 1991 On the use of mathematical models of malaria transmission. Acta Trop. 49, 1–25. (10.1016/0001-706X(91)90026-G) 1678572

[30] Hairston NG, de Meillon B 1968 On the inefficiency of transmission of *Wuchereria bancrofti* from mosquito to human host. Bull. World Health Organ. 38, 935–941. 4235739 PMC2554522

[31] Carter R, Mendis KN, Roberts D 2000 Spatial targeting of interventions against malaria. Bull. World Health Organ. 78, 1401–1411. 11196487 PMC2560653

[32] Bousema T, Baidjoe A 2013 Heterogeneity in malaria transmission: underlying factors and implications for disease control. In *Ecology of parasite-vector interactions* (eds W Takken, CJM Koenraadt), pp. 197–220. Berlin, Germany: Springer.

[33] Bockarie MJ, Tisch DJ, Kastens W, Alexander NDE, Dimber Z, Bockarie F, Ibam E, Alpers MP, Kazura JW 2002 Mass treatment to eliminate filariasis in Papua New Guinea. N. Engl. J. Med. 347, 1841–1848. (10.1056/NEJMoa021309) 12466508

[34] Bockarie M, Kazura J, Alexander N, Dagoro H, Bockarie F, Perry R, Alpers M 1996 Transmission dynamics of Wuchereria bancrofti in East Sepik Province, Papua New Guinea. Am. J. Trop. Med. Hyg. 54, 577–581. (10.4269/ajtmh.1996.54.577) 8686774

[35] Reimer LJ *et al.* 2013 Insecticidal bed nets and filariasis transmission in Papua New Guinea. N. Engl. J. Med. 369, 745–753. (10.1056/NEJMoa1207594) 23964936 PMC3835352

[36] Reimer LJ, Thomsen EK, Koimbu G, Keven JB, Mueller I, Siba PM, Kazura JW, Hetzel MW, Zimmerman PA 2016 Malaria transmission dynamics surrounding the first nationwide long-lasting insecticidal net distribution in Papua New Guinea. Malar. J. 15, 25 (10.1186/s12936-015-1067-7) 26753618 PMC4709896

[37] R Core Team. R: a Language and environment for statistical computing. Vienna, Austria: R Foundation for Statistical Computing.

[38] Rue H, Martino S, Chopin N 2009 Approximate Bayesian inference for latent Gaussian models by using integrated nested laplace approximations. J. R. Stat. Soc. B Stat. Methodol. 71, 319–392. (10.1111/j.1467-9868.2008.00700.x)

[39] Martins TG, Simpson D, Lindgren F, Rue H 2013 Bayesian computing with INLA: new features. Comput. Stat. Data Anal. 67, 68–83. (10.1016/j.csda.2013.04.014)

[40] Johnson JB, Omland KS 2004 Model selection in ecology and evolution. Trends Ecol. Evol. 19, 101–108. (10.1016/j.tree.2003.10.013) 16701236

[41] World Health Organization *et al.*. 2011 *Monitoring and epidemiological assessment of mass drug administration in the global programme to eliminate lymphatic filariasis: a manual for national elimination programmes*. Geneva, Switzerland: WHO.

[42] Hasibeder G, Dye C 1988 Population dynamics of mosquito-borne disease: persistence in a completely heterogeneous environment. Theor. Popul. Biol. 33, 31–53. (10.1016/0040-5809(88)90003-2) 2897726

[43] Lloyd AL, Zhang J, Root AM 2007 Stochasticity and heterogeneity in host–vector models. J. R. Soc. Interface 4, 851–863. (10.1098/rsif.2007.1064) 17580290 PMC2394551

[44] Perkins TA, Scott TW, Le Menach A, Smith DL 2013 Heterogeneity, mixing, and the spatial scales of mosquito-borne pathogen transmission. PLoS Comput. Biol. 9, e1003327 (10.1371/journal.pcbi.1003327) 24348223 PMC3861021

[45] Rao RU, Nagodavithana KC, Samarasekera SD, Wijegunawardana AD, Premakumara WDY, Perera SN, Settinayake S, Miller JP, Weil GJ 2014 A comprehensive assessment of lymphatic filariasis in sri lanka six years after cessation of mass drug administration. PLoS Negl. Trop. Dis. 8, e3281 (10.1371/journal.pntd.0003281) 25393404 PMC4230885

[46] Small ST, Reimer LJ, Tisch DJ, King CL, Christensen BM, Siba PM, Kazura JW, Serre D, Zimmerman PA 2016 Population genomics of the filarial nematode parasite *Wuchereria bancrofti* from mosquitoes. Mol. Ecol. 25, 1465–1477. (10.1111/mec.13574) 26850696 PMC4808423

[47] World Health Organization 2017 Summary of global update on preventive chemotherapy implementation in 2016: crossing the billion. Wkly. Epidemiol. Rec. 92, 589–608. 28984120

[48] Vazquez-Prokopec GM, Perkins TA, Waller LA, Lloyd AL, Reiner RC, Scott TW, Kitron U 2016 Coupled heterogeneities and their impact on parasite transmission and control. Trends Parasitol. 32, 356–367. (10.1016/j.pt.2016.01.001) 26850821 PMC4851872

[49] Michael E, Bundy DAP 1998 Herd immunity to filarial infection is a function of vector biting rate. Proc. R. Soc. London B: Biol. Sci. 265, 855–860. (10.1098/rspb.1998.0370) PMC1689054 9633111

[50] Michael E, Malecela-Lazaro MN, Kabali C, Snow LC, Kazura JW 2006 Mathematical models and lymphatic filariasis control: endpoints and optimal interventions. Trends Parasitol. 22, 226–233. (10.1016/j.pt.2006.03.005) 16564745

