# Supplementary material for: Understanding heterogeneities in mosquito-bite exposure and infection distributions for the elimination of lymphatic filariasis
Source: Proc Biol Sci. 2018 Jan 31;285(1871):20172253. doi: 10.1098/rspb.2017.2253 (PMC5805933; doi:10.1098/rspb.2017.2253)
Supplement: Supplementary information [file rspb20172253supp1.pdf]

Understanding heterogeneities in mosquito-bite exposure and infection distributions for the elimination of lymphatic filariasis: supplementary information

Michael A Irvine[1,2], James W Kazura[3], T Deirdre Hollingsworth[1,4], and Lisa J Reimer[5]

[1]School of Life Sciences, University of Warwick, UK
[2]Institute of Applied Mathematics, University of British Columbia, Vancouver, Canada
[3]Center for Global Health and Disease, Case Western Reserve University, Cleveland, USA
[4]Big Data Institute, Li Ka Shing Centre for Health Information and Discovery, University of Oxford, Oxford, UK
[5]Department of Vector Biology, Liverpool School of Tropical Medicine, Liverpool, UK

January 30, 2018

Fixed effect coefficients

The fixed effect regression coefficients for the single spatial models are laid out in tables 2 to 5. These include their intercept, sex of individual where applicable, age of individual where applicable and the maximum coefficient of the breeding site. Only age was found to be consistently statistically significant when modelling infection status ($p < 0.001$), although the size of the effect was small. when predicting the spatial distribution of nightly bites the presence of bed-nets was found to be statistically significant (table 5).

Regression coefficients for the combined models are also given (table 6). All coefficients were found to be statistically significant.

Spatial kernel coefficients

The fitted spatial coefficients for the mf count model were compared to the number of nightly bites model (fig. 3). The Matern covariance function can be parameterised by the practical range, variance and the kappa. The practical range for the bites was found to be greater than for the mf count, although there was significant overlap in the marginal posteriors (fig. 3a). The variance was found to be significantly smaller for the bites compared to mf count (fig. 3b), and there was not found to be significant difference in the kappa (fig. 3c). The other infection status spatial coefficients behaved similarly to the mf count fitted covariance.

Distance to breeding site

The distance to the nearest breeding site was explored as a potential explanatory variable for infection status and nightly number of bites (fig. 4a). An exploratory analysis showed a weak relationship between nightly bites and distance. As such a one-dimensional random walk was used to model the distance from breeding site (fig. 4b). For all models, distance to breeding site was found not to be statistically significant as, although there is a general negative trend

as distance increases, the 95% confidence intervals include zero for all, but a few extreme points where there is little data.

Supplementary tables

Model	DIC
Ag only	950.73
mf count only	5330.19
mf prevalence only	793.20
Ag combined with bites	9874.19
mf count combined with bites	13044.11
mf prevalence combined with bites	9631.25

Table 1: Deviance Information Criterion for all six spatial models

	mean	sd	p-value
Intercept	-2.26	1.18	0.05
Sex (M)	0.26	0.18	0.15
Age	0.034	0.0052	< 0.001
Distance to breed site (max. coefficient)	8.05×10^{-6}	0.00549	1.00

Table 2: Fixed effect regression coefficients for microfilaraemia spatial model

	mean	sd	p-value
Intercept	-0.75	0.82	0.36
Sex (M)	0.32	0.16	0.05
Age	0.041	0.0050	< 0.001
Distance to breed site (max. coefficient)	1.17×10^{-4}	0.015	0.99

Table 3: Fixed effect regression coefficients for antigenaemia spatial model

	mean	sd	p-value
Intercept	2.39	1.4	0.08
Sex (M)	1.10	0.45	0.02
Age	0.062	0.012	< 0.001
Distance to breed site (max. coefficient)	4.60×10^{-5}	0.016	1.00
k	0.050	0.0043	NA

Table 4: Fixed effect regression coefficients for mf count spatial model

	mean	sd	p-value
Intercept	3.19	0.62	< 0.001
LLIN	-1.86	0.10	< 0.001
Distance to breed site (max. coefficient)	1.50	2.11	0.47
k	0.62	0.028	NA

Table 5: Fixed effect regression coefficients for nightly bites spatial model

	mean	credible interval
mf count	1.71	(1.38, 2.06)
microfilaraemia	1.00	(0.96, 1.00)
antigenaemia	1.17	(1.13, 1.21)

Table 6: Strength of bites field on predicting the mf count, microfilaraemia and antigenaemia. The bite distribution appears best at predicting the antigenaemia and less well at the count itself. This indicates that distribution of bites may indicate prevalence but may not necessarily indicate intensity.

Supplementary figures

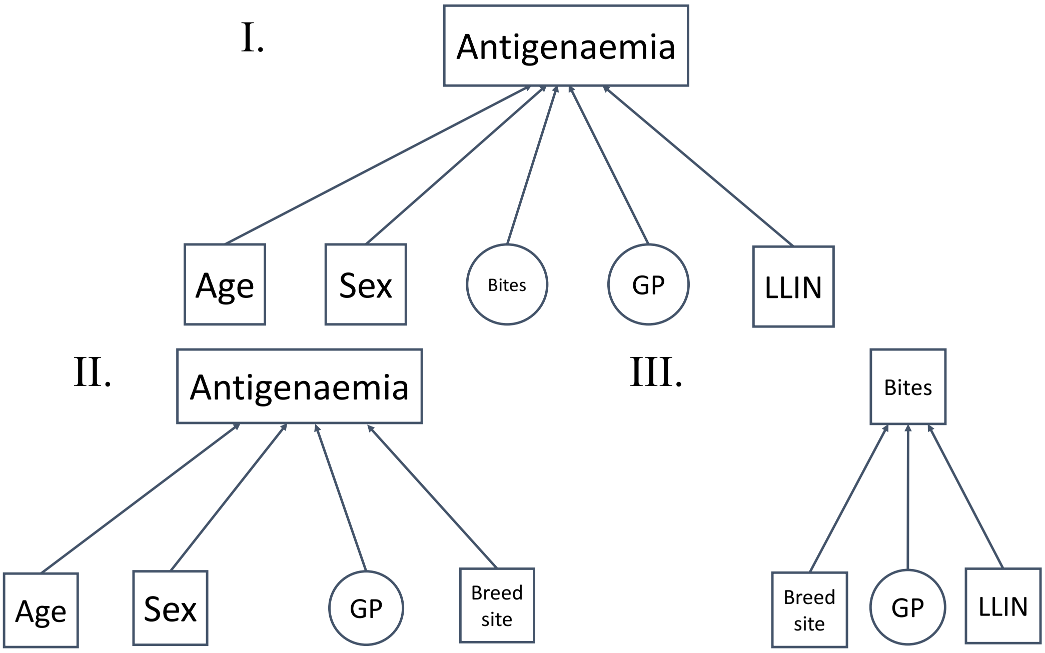

Figure 1: Summary of model structures used in spatial analysis. Model I is the combined model, where Antigenaemia (or microfilaraemia or mf density) is dependent upon age, sex, presence of LLIN, as well as a spatial field fitted to mosquito bite data and a fitted Gaussian process (GP) representing the spatial variation in the outcome measure as well as a non-spatial error term. Model II is an infection status outcome measure, which is dependent on age, sex, distance to breeding site as well as a general Gaussian process spatial field and an error term. Model III is the nightly mosquito bite model dependent on distance to breeding site, presence of LLIN and a Gaussian process representing the underlying spatial variation. Parameters in circles have spatial dependency and square parameters do not.

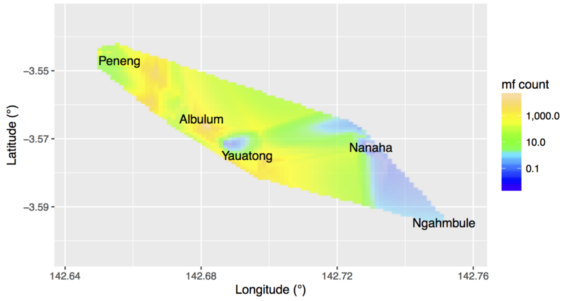

(a)

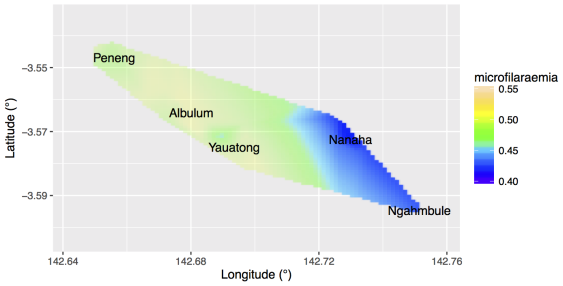

(b)

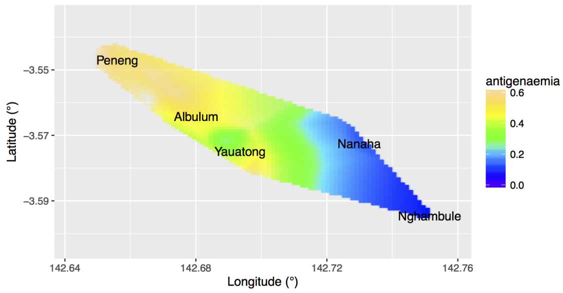

(c)

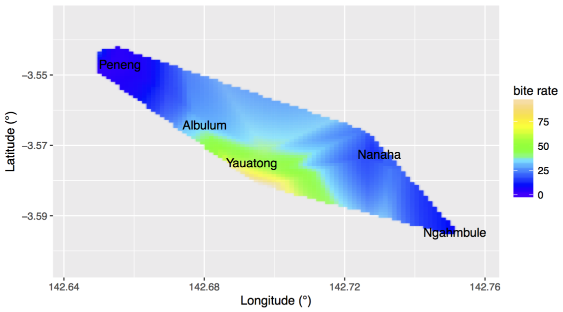

(d)

Figure 2: Spatial fits of hierarchical model including all known covariates including distance to breeding site. The four outcome measures that were fitted are: **(a)** mf count **(b)** mf prevalence **(c)** antigen prevalence and **(d)** nightly bite rate. The uncertainty in the estimate is shown as the transparency in the colour plot, for example although the appears that microfilaremia prevalence is highest around 3.55°S,142.68°W, this is where the standard deviation in the estimate is at its highest.

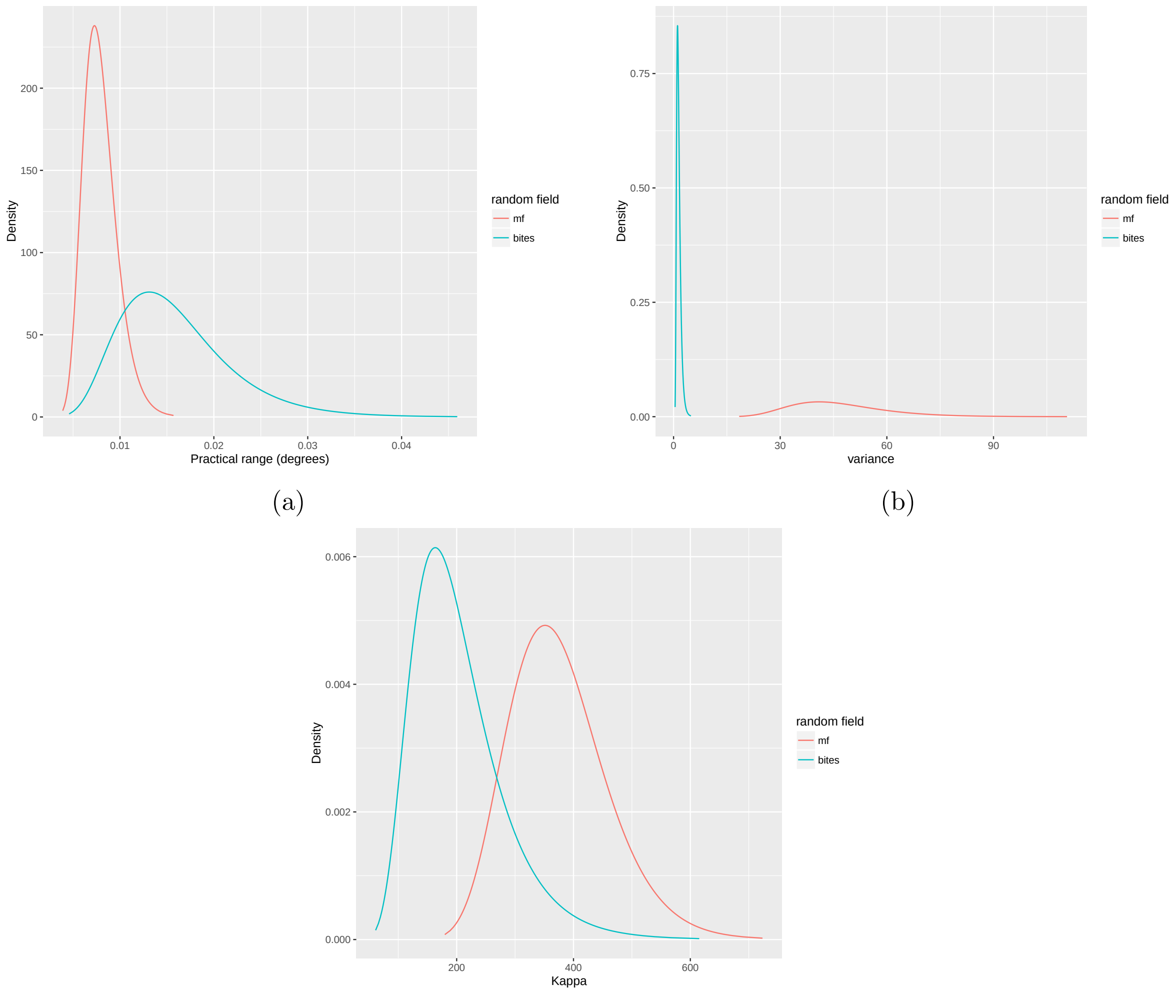

(a) (b) (c)

Figure 3: Comparison between fitted Matern covariance functions for combined mf/bites model. The practical range indicates that the spatial correlation between bites is greater than that for the mf. The non-spatial variance indicates that the bites field is more strongly dependent on space, whereas there is a lot of variation that is not captured by spatial effects for the mf distribution.

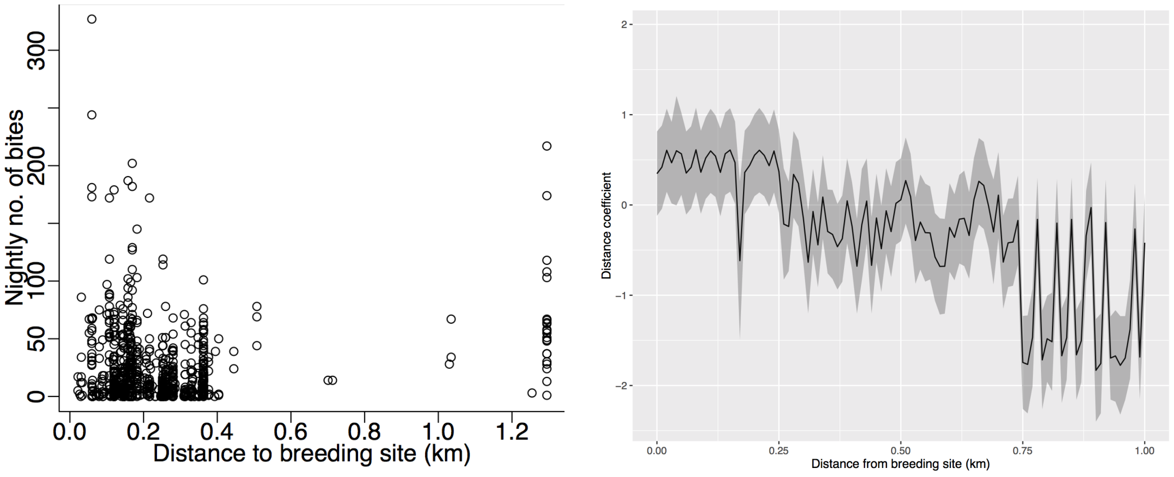

(a) (b)

Figure 4: **(a)** Relationship between distance to nearest breeding site and number of nightly bites. As can be observed there is a non-linear relationship between the two exacerbated by the data being negative-binomially distributed. Instead of using a standard linear covariate, the covariate for the underlying latent field may be modelled as a random walk of dimension one with change in displacement drawn from a normal distribution with zero mean. **(b)** Strength of distance to breeding site coefficient to predict distribution of bites. Instead of using a standard linear covariate, the covariate for the underlying latent field may be modelled as a random walk of dimension one with change in displacement drawn from a normal distribution with zero mean. Although there is a general downward trend in strength, the confidence intervals include the zero and hence is shown to not be significant.

Figure 5

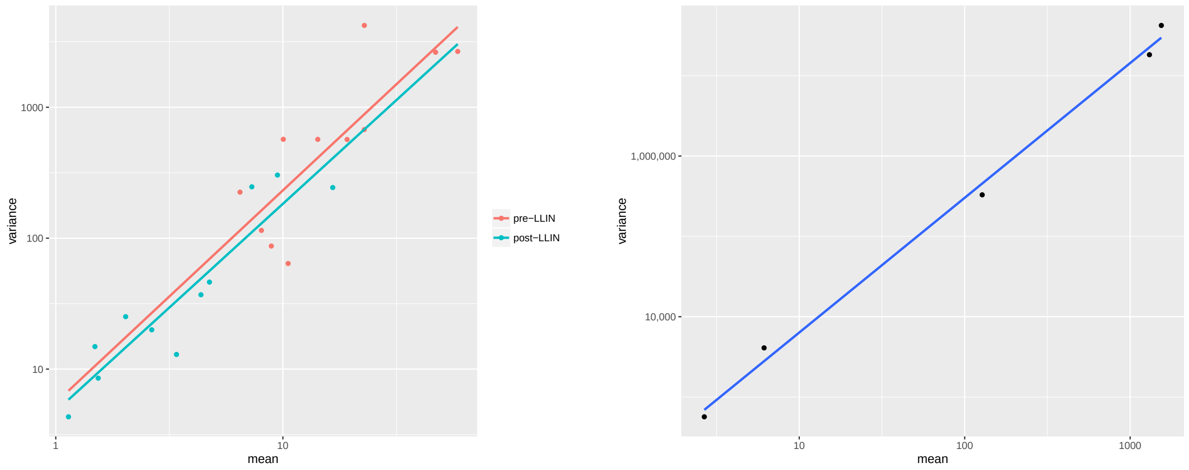

(a) (b)

Figure 6: Mean-variance relationships. **(a)** mean-variance relationship for the village nightly bites, separated before and after bed-nets. **(b)** mean-variance relationship for the mf count amongst villages.
